# Application of the respiratory “critical care-sub-critical care-rehabilitation integrated management model” in severe stroke associated pneumonia

**DOI:** 10.1186/s12890-020-1100-7

**Published:** 2020-03-05

**Authors:** Xue-Lin Wang, Li-Jun Ma, Xin-Gang Hu, Kai Wang, Jian-Jian Cheng

**Affiliations:** Department of Respiratory and Critical Care Medicine, Henan Provincial People’s Hospital, People’s Hospital of Zhengzhou University, People’s Hospital of Henan University, No. 7 of Weiwu Road, Jinshui District, Zhengzhou, 450003 Henan China

**Keywords:** Respiratory “Critical Care-Sub-critical Care-Rehabilitation Integrated Management Model”, Stroke-associated pneumonia, Severe pneumonia

## Abstract

**Background:**

This study aimed to explore the feasibility of applying the respiratory “critical care-sub-critical care-rehabilitation integrated management model” in severe stroke-associated pneumonia and evaluate its effect.

**Methods:**

From January to September 2018, 24 patients with severe stroke-associated pneumonia, who were admitted to the Respiratory Intensive Care Unit of the Respiratory and Critical Care Medicine Department of Henan Provincial People’s Hospital, were randomly divided into two groups: integrated management group and control group. According to the admission criteria of the respiratory “critical care-sub-critical care-rehabilitation integrated model” prescribed by the above-mentioned hospital, patients were grouped. The professional respiratory therapy team participated in the whole treatment. The acute physiology and chronic health evaluation II (APACHE II) score, clinical pulmonary infection score (CPIS) and oxygenation index of these two groups were dynamically observed, and the average hospital stay, 28-day mortality and patient satisfaction were investigated.

**Results:**

Patients in the integrated management group and control group were similar before treatment (*P* > 0.05). After treatment, the main indicators, the APACHE II score, CPIS score and oxygenation index, were significantly different between the integration group and control group (*P* < 0.05). The secondary indicators, the average hospitalization days and patient/family member satisfaction scores, were also significantly different between the integration group and control group (*P <* 0.05). However, the 28-day mortality wasn’t significantly different (*P* > 0.05).

**Conclusions:**

For patients with severe stroke-associated pneumonia, it was feasible to implement the respiratory “critical care-sub-critical care-rehabilitation integrated management model”, which could significantly improve the treatment effect, shorten average hospitalization days and improve patient/family satisfaction.

## Background

Stroke-associated pneumonia (SAP) is one of the most common in-hospital medical complications after stroke [1]. Pneumonia is a key risk factor for stroke death, which has an incidence of 7–22% [2]. It couldn’t be addressed before pneumonia occurs due to the present policy implementation which could leads to a sharp increase in medical costs [3]. The mortality rate of severe pneumonia (SP) can reach up to 17–55% [4–8], and severe SAP may have a higher mortality rate. However, there is no exact statistical data on this at present.

Clinically, severe SAP patients have the following characteristics: (1) Disease: the onset age is usually advanced, the recovery time of the primary disease is long, the number of complications is large, and the condition is prone to recurrence and aggravation; (2) Treatment professional team: the requirements for the professional treatment team, especially in respiratory support therapy and respiratory rehabilitation, are high; (3) Management: there are high requirements for multidisciplinary treatment, cooperation between doctors, nurses, rehabilitation therapists and patients, and smooth convergence between treatment and rehabilitation. Besides, increasing pneumonias associated with stroke are admitted to the study hospital, which is the largest comprehensive hospital in Henan. Patients from the local and surrounding five provinces will stay in our hospital, resulting in a sharp increase in the number of patients of pneumonias associated with stroke. However, the current situation of severe patients in respiratory medicine includes the particularity of the disease, insufficient cooperation of professional teams, and poor management cohesion. The final result is that patients have to stay in hospital for a long time and have a high rate of reexamination. High mortality rate, high average hospitalization cost and low satisfaction of patients’ family members need comprehensive management model to improve treatment efficiency and quality.

Based on this situation, the investigators integrated resources in the Department of Respiratory and Critical Care Medicine to use the respiratory “critical care-sub-critical care-rehabilitation integrated management model”, and compared this with the present department-based management model, in order to explore the feasibility of the integrated management model and evaluate its effect.

## Methods

### Study design, setting and patients recruitment

**Fig. 1:**
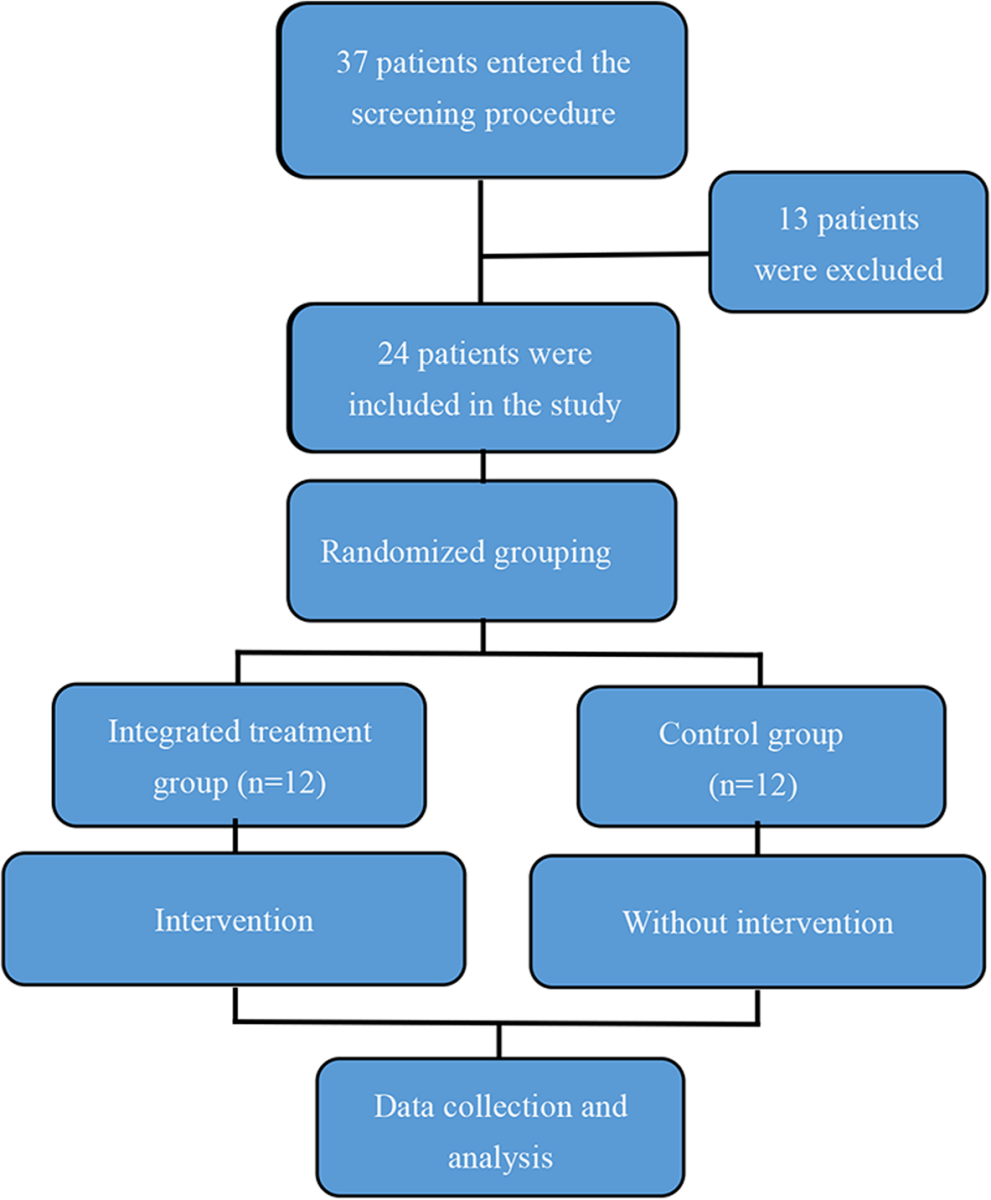
Study flow of the recruitment and analysis process

This study was a single center and a randomized feasibility trial. Study patients, who were admitted to the Respiratory Intensive Care Unit (RICU) of the Department of Respiratory and Critical Care Medicine, Henan Provincial People’s Hospital, China from January to September 2018 due to severe SAP, were enrolled into the present study. They were recruited by means of researcher’s initiative introduction, relevant departments’ proposal and recruitment advertisement. All patients met the criteria for SAP [8] and severe pneumonia [9]. These patients were randomly assigned either to the integrated management group (intervention group) or to no intervention/usual care (control group) by simple random number method. The number recruited, randomized, fail to followed-up and number for analysis was shown in Fig. 1.

Eligible participants were identified from the local large medical-center-Henan Provincial People’s Hospital. All patients suffering from pneumonia admitted to the center were screened for study eligibility by the physician or deputy Chief physician of the research team. Eligible patients were consecutively informed about the study and invited to participate. When signing the informed consent, the patient and his/her family members had been informed in detail of the following contents: the purpose of the study, how to cooperate with the implementation of the study, possible benefits and risks, the rights and interests of the patient, the confidentiality of information and the freedom to participate and withdraw. The rejection rate of patients was less than 30%, target characteristics were consistent. After written informed consent, randomized grouping and baseline assessments were conducted. This study was conducted in accordance with the declaration of Helsinki and approved by the Ethics Committee of Henan Provincial People’s Hospital.

#### Diagnostic criteria of SAP and SP

Diagnostic criteria of SAP: Excluding pulmonary infection before stroke, showing patchy shadows by pulmonary X-ray examination after stroke, and at least two of the four items must be included: (1) presence of cough, expectoration or worsening of original respiratory symptoms with or without chest pain; (2) fever (> 38 °C); (3) Signs of lung consolidation and / or moist rales; (4) white blood cell ((WBC > 10 × 10^9^/L or < 4 × 10^9^/L)) in peripheral blood (WBC > 10 × 10^9^/L or < 4 × 10^9^/L) with or without left shift of nucleus.

Diagnostic criteria of SP: The Chinese adult SAP guidelines in 2015 also adopt new simplified diagnostic criteria: those who meet the following one main criteria or more than three secondary criteria can be diagnosed as SP, and need close observation and active treatment, and it is recommended that they accept treatment in intensive care unit (ICU). Main criteria: (1) mechanical ventilation is needed for tracheal intubation; (2) vasoactive drugs are still needed after active fluid resuscitation for septic shock. Secondary criteria: (1) respiratory rate > 30 times per minute; (2) arterial oxygen tensio/Inspired oxygen fraction (PaO_2_/FiO_2_) < 250 mmHg; (3) infiltration on chest x-ray; (4) disturbance of consciousness and/or orientation; (5) blood urea nitrogen (> 7 mmol/L); (6) hypotension requiring active fluid resuscitation.

The diagnosis of severe SAP in admitted patients was determined by the Chief Physician or deputy Chief Physician of the research team.

#### Inclusion criteria and exclusion criteria

Inclusion criteria: meet the diagnostic criteria of SAP and severe pneumonia; meet the ICU admission criteria; age range from 18 to 75 years old; informed consent should be signed by patients or family members.

Exclusion criteria: severe irreversible organ dysfunction such as heart, liver and kidney; patients who were participating in other experimental studies; researchers considered that any reason was not suitable for participating in this trial, such as poor economic basis or poor communication ability who would be most probable to withdraw, and medical dispute tendency, which was determined by the researchers who are specially in charge of case enrollment. These researchers are responsible for talking and signing the informed consent. In order to avoid sample bias, these researchers had excluded those with poor economic foundation during the recruitment process.

### Intervention

#### The components of the integrated management model

In the control group, the direct referral between the RICU and General Ward was carried out. According to the standard requirements of the integrated management model, patients in the integrated management group were strictly managed in accordance with the management procedures of respiratory “critical care-sub-critical care-rehabilitation integration”. The model group received additional early and follow-up rehabilitation exercises and correct rehabilitation education than the control group.

#### The admission criteria for the intervention

The admission criteria were as followed: (1) situations requiring invasive or non-invasive mechanical ventilation induced by respiratory diseases, such as SP, acute lung injury (ALI) / acute respiratory distress syndrome (ARDS), acute exacerbations of chronic obstructive pulmonary disease (AECOPD), severe asthma, pulmonary embolism, lung tumors and interstitial lung disease, acute respiratory failure, or acute exacerbation of chronic respiratory insufficiency; (2) secondary respiratory insufficiency requiring the support of respiratory or other important organs induced by other systemic diseases, such as cardiac dysfunction or neuromuscular dysfunction, surgery, such as lung transplantation and other thoracic surgery at the perioperative period and post-cardiopulmonary resuscitation period, and particularly situations that require invasive or non-invasive mechanical ventilation; (3) situations where hemodynamics is instable, and there is a requirement for vasoactive drugs, external counterpulsation and other treatments; (4) situations where organ functions are degraded and respiratory function may be affected. (A) Admission range of the RICU: patients who met one of the following situations: (1) + (3), (1) + (4), (2) + (3), or (2) + (4); (A) Admission range of sub-critical RICU: patients who met (1) or (2) and were excluded from (4); (A) Admission range of respiratory the rehabilitation ward: patients who met (1), and turned to this ward with the purpose of pulmonary rehabilitation after critical/sub-critical intensive care and treatment, and patients with stable conditions who met (2), (3) and (4).

#### Establishment of a respiratory treatment team

A respiratory treatment team that consisted of a respiratory physician as the leader and eight respiratory technicians was established. This team participated in the education and guidance during the whole process of the critical respiratory support, the early stage rehabilitation of sub-critical pulmonary disease and pulmonary rehabilitation in the rehabilitation ward. All staffs included physicians, rehabilitation technicians and nurses were trained in projects requirements before study onset.

#### RICU

This mainly targeted patients with primary critical respiratory diseases and hemodynamic instability, where support therapies for critical respiratory diseases, such as extracorporeal membrane oxygenation (ECMO) and invasive mechanical ventilation, were conducted, in order to allow patients to early transfer to the sub-critical ward for early rehabilitation and to improve organ functions.

#### Sub-critical RICU

This unit mainly targeted patients with primary critical respiratory diseases and hemodynamic stability, and early pulmonary rehabilitation treatments, such as weaning screening, weaning test, chest physical therapy and neuromuscular function training, were carried out to help patients turn to the rehabilitation unit for transitional treatment as soon as possible.

#### Respiratory rehabilitation ward

This mainly targeted patients with respiratory failure, who didn’t need intensive care, in which pulmonary rehabilitation treatments, such as non-invasive mechanical ventilation, open airway management and continuous respiratory exercise, were carried out for the purpose of providing treatment for patients to obtain good organ function recovery, and providing technical guidance for the home treatment of patients.

#### Patient/family satisfaction evaluation system

Based on the contents of the inpatient satisfaction evaluation system developed by Yan Liu et al. [10–12], a patient/family satisfaction survey questionnaire was developed by relevant responsible personnel with the main contents of the evaluation of all aspects of doctors and nursing works. The Likert five-point score scale was adopted.

#### Sample size, randomization and blinding procedures

The sample size calculation was based on the lung function and shorten RICU stay. For the estimation of sample size, four factors are set: α = 0.05, β = 0.20 (Power = 0.80), σ = 50 and δ = 30. At least 12 subjects are required for each group. The hypothesis was improvement in the intervention group and maintenance in control group. The well-established minimal important difference of the oxygenation index was 80. Assuming a standard deviation of the outcome variable of 70–80% power and a significance level of 0.05 (two-sided), a sample size of 12 patients in each group was required which resulted in a total sample size of 24. Because intervention and data collation were done in the hospital, so drop-out was not considered.

Patients were randomized separately, on the level of patients, using random number table with a 1:1 ratio. An independent biostatistician developed a separate randomization list using random number table. Another independent researcher implemented these lists into the database. When the baseline were all assessed, randomization could be applied.

In consideration of the intervention process, both study patients and health care professionals couldn’t be blinded after the patients’ assignment to the intervention or control group. Data analysts was blinded to group assignment.

#### Data management

The data of this experiment were collected in hospital and followed up by telephone after discharge. For the model group special researchers were arranged to collect data, which was feasible. The observed test indexes are consistent with those reported in the research literature, and have good sensitivity. No new test indexes need to be formulated.

### Observation indexes

Main observation indexes: acute physiology and chronic health evaluation II (APACHE II) score, clinical pulmonary infection score (CPIS) and oxygenation index [13–15]. The APACHE II score, CPIS score and oxygenation index were assessed per 24–48 h. The data within 24 h of the patient’s admission to the RICU was recorded as baseline, while the data at the time of discharge from the hospital was recorded to evaluate the efficacy. Secondary observation indexes: average hospitalization days, 28-day mortality rate and patient/family satisfaction rate. As a relatively objective indicator of effectiveness of treatment by reassigning questionnaires to patients or their family members.

### Statistics analysis

Data analysis was conducted using the statistical software SPSS (version 26; IBM Corp, Armonk, New York, USA). Measurement data were compared using *t*-test. Count data were compared using *X*^2^-test of two samples. The significance level was α = 0.05.

## Results

### Patients recruitment

The study flow was showed in Fig. 1.

### Acceptability and suitability of intervention and study procedures

In the course of research intervention and follow-up, the subjects had good coordination and compliance, and the period from discharge to follow-up was short. Because patients who died during the trial were recorded and all others were followed by telephone, thus the retention rate and follow-up rate reached 100%. No intentional adverse events occurred.

### Comparison of basic conditions between the integrated management group and control group

The comparative analysis revealed that the differences in all baseline indexes between these two groups were not statistically significant (*P* > 0.05). This suggests that the basic conditions of these two groups were similar. Hence, these two groups were comparable (Table 1).

**Table 1:** Comparison of basic conditions between the integrated management group and control group

Group	Integrated management group	Control group	*P value*
Case (n)	12	12	
Age (years)	66.67 ± 9.48	67.42 ± 10.81	0.858
Gender (male/female)	7/5	8/4	0.680
Tracheal intubation and mechanical ventilation (n)	5	4	0.680
Oxygenation index (mmHg)	174.08 ± 44.19	146.75 ± 45.04	0.148
CPIS score	9.92 ± 1.24	9.58 ± 1.08	0.491
APACHE II score	24.83 ± 6.89	27.17 ± 6.69	0.409

*CPIS score* clinical pulmonary infection score, *APACHE II score* acute physiology and chronic health evaluation II score

### Comparison of main observation indexes between the integrated management group and control group

After treatment, the differences in APACHE II score, CPIS and oxygenation index between the integrated management group and control group were statistically significant (*P* < 0.05, Table 2). This suggested that integrated management could improve the effect of severe SAP.

**Table 2:** Comparison of treatment effect and prognosis between the integrated management group and control group

Group	Integrated management group	Control group	*P value*
Case (n)	12	12	
Oxygenation index (mmHg)	308.50 ± 82.51	231.25 ± 60.80	0.017
CPIS score	3.08 ± 2.23	5.17 ± 2.44	0.040
APACHE II score	14.83 ± 3.13	18.92 ± 5.73	0.045
mean hospitalization day (day)	20.67 ± 4.19	25.00 ± 5.01	0.032
28-day mortality rate (n, %)	1 (8.33)	3 (25.0)	0.284
Patients/family’s satisfaction rate	93.08 ± 4.46	88.67 ± 4.10	0.019

*CPIS score* clinical pulmonary infection score, *APACHE II score* acute physiology and chronic health evaluation II score

### Comparison of secondary observation indexes between the integrated management group and control group

After treatment, among the secondary observation indexes, the differences in mean hospitalization day and satisfaction rate of patients/family members between the integrated management group and control group were statistically significant (*P* < 0.05). However, the difference in 28-day mortality rate wasn’t statistically significant (*P* > 0.05, Table 2). This suggested that integrated management could shorten the mean hospitalization stay and improve patient/family satisfaction rate in severe SAP. However, this couldn’t significantly reduce the 28-day mortality rate.

## Discussion

In this study, “critical care-sub-critical care-rehabilitation integrated management model” was proposed and the feasibilities were identified. This integrated management model addressed three essential problems. First, this intervention could shorten the hospitalization stay, which eased the pressure of hospital and doctor. Besides, for the family of patients with severe stroke-associated pneumonia, the economic burden was decreased. Finally, what should be highlighted in this study was that the integrated management model could improve the treatment effect of patients with severe SAP, which provided a more feasible method to more patients with severe SAP.

The integrated management model was accessible and feasible, because of the following reasons: under the background of the current economic level and the “expensive medical treatment” caused by the medical system and the national government’s policy of advocating graded diagnosis and treatment, hospital management attaches great importance and supports. Medical and nursing staff possess extraordinary professionalism. Furthermore, adequate source of patients could be obtained. The management model was easy to be accepted by stakeholders and had a high retention rate: the national health insurance policy had been significantly improved, the reasonable diagnosis of patients was guaranteed by important policy documents in the hospital, the cooperation of relevant departments and the compliance of patients is good. In addition, by consulting the cost of some patients and estimating the number of days in hospital, the average cost of hospitalization of patients after intervention is lower than that of non-intervention group.

In addition, the investigators have drafted and formulated the criteria for the treatment of the integrated management model, defined the treatment scope, treatment features and treatment objectives of the critical wards for respiratory, sub-critical respiratory and rehabilitation, and the patients were treated and transferred strictly according to the admission criteria, in order to ensure the safety and homogeneity of these patients. Furthermore, the integrated management model would require that each patient is managed by the same physician-in-charge, in order to ensure the continuity of the patient’s treatment and help improve the satisfaction rate. Finally, if a professional respiratory treatment team is established, which is consisted of senior respiratory physicians and several respiratory rehabilitation technicians, it can provide more evidence about how the treatment effect of integrated management model could be maintained or raised in the long term. It is worth noting that these staff receive experience unified training. They participate in the whole process of the critical respiratory support, sub-critical pulmonary early rehabilitation and pulmonary rehabilitation in the rehabilitation wards. Through the above-mentioned measures, the smooth implementation of the integrated model could be ensured.

This integrated management model had a good effect in the clinical treatment of severe SAP. Pulmonary ventilation and/or ventilation function is decreased in patients with severe SAP. The arterial partial pressure of oxygen was lower than the normal range, with or without an increase in partial pressure of carbon dioxide. These suggested that these patients had respiratory dysfunction, which showed the importance of rehabilitation in decreasing mortality rate and prolonged length of hospital stay [16]. Hence, this should be assessed and intervened with pulmonary rehabilitation as soon as possible [17]. Pulmonary rehabilitation [18], especially early intensive pulmonary rehabilitation, is an important link in the respiratory management of patients. For critical patients with mechanical ventilation [19, 20], patients with inadequate nutritional support [21], or patients with myasthenia induced by hormones and other drugs [22, 23] as the use of a home ventilator increases, patients with long-term ventilator support and some patients with long-term ventilator support caused by difficult weaning have significant surrounding skeletal muscle atrophy [24, 25]. Hence, intensive pulmonary rehabilitation is helpful in improving their condition and shortening the weaning process.

## Conclusion

In this study, the investigators concentrated closely on the objectives of a feasibility study. By investigating these five objectives, the investigators ensured that whether this study could be processed. It could be concluded that the application of the integrated model in patients with severe SAP could improve the effect by improving the APACHE II score, CPIS score and oxygenation index of patients, and shorten the mean hospitalization stay of patients, thereby improving the patient/family satisfaction rate.

## Data Availability

The datasets generated and/or analysed during the current study are not publicly available due to the lack of an online platform but are available from the corresponding author on reasonable request.

## Abbreviations

AECOPD: Acute exacerbations of chronic obstructive pulmonary disease
ALI: Acute lung injury
APACHE II: Acute physiology and chronic health evaluation II
ARDS: Acute respiratory distress syndrome
CPIS: Clinical pulmonary infection score
ECMO: Extracorporeal membrane oxygenation
ICU: Intensive care unit
RICU: Respiratory Intensive Care Unit
SAP: Stroke-associated pneumonia
SP: Severe pneumonia
